# Motion style acupuncture treatment (MSAT) for acute low back pain with severe disability: a multicenter, randomized, controlled trial protocol

**DOI:** 10.1186/1472-6882-11-127

**Published:** 2011-12-13

**Authors:** Joon-Shik Shin, In-Hyuk Ha, Tae-Gyu Lee, Youngkwon Choi, Byoung-Yoon Park, Me-riong Kim, Myeong Soo Lee

**Affiliations:** 1 Jaseng Medical Foundation, Jaseng Hospital of Oriental Medicine, Seoul, Republic of Korea; 2 Division of Standard Research, Korea Institute of Oriental Medicine, Daejeon, Republic of Korea

## Abstract

**Background:**

Acupuncture is widely-used to treat patients with low back pain, despite insufficient evidence of the technique's efficacy for acute back pain. Motion style acupuncture treatment (MSAT) is a non-traditional acupuncture treatment requiring a patient to exercise while receiving acupuncture. In Korea, MSAT is used to reduce musculoskeletal pain and improve functional status. The study aims to evaluate the effect of MSAT on acute low back pain with severe disability.

**Methods/Design:**

This study is a multicenter, randomized, active-controlled trial with two parallel arms. Participants with acute low back pain and severe functional disability, defined as an Oswestry Disability Index (ODI) value > 60%, will be randomly allocated to the acupuncture group and the nonsteroidal anti-inflammatory drug (NSAID) injection group. The acupuncture group will receive MSAT and the NSAID injection group will receive an intramuscular injection of diclofenac. All procedures will be limited to one session and the symptoms before and after treatment will be measured by assessors blinded to treatment allocation. The primary outcome will be measured at 30 minutes after treatment using the numerical rating scale (NRS) of low back pain while the patient is moving. Secondary outcomes will be measured at 30 minutes after treatment using the NRS of leg pain, ODI, patient global impression of change, range of motion (ROM) of the lumbar spine, and degrees of straight leg raising (SLR). Post-treatment follow-up will be performed to measure primary and secondary outcomes with the exception of ROM and SLR at 2, 4, and 24 weeks after treatment.

**Discussion:**

The results of this trial will be discussed.

**Trial Registration:**

ClinicalTrial.gov NCT01315561

## Background

Low back pain is a common symptom that greatly impacts individuals and societies and is experienced by 70-80% of adults at least once in their lives [1]. Approximately $26.3 billion was spent due to low back pain in the United States in 1998 [2]. Because back pain is usually a self-limiting and benign condition, patients who experience acute back pain typically see improvements and are able to return to work within a month [3,4]. However, 2-7% of patients develop chronic back pain, and 75-85% of absences from work are due to chronic or recurrent back pain [5,6]. Thus, when acute back pain occurs, it is important to reduce it using a treatment with minimal side effects to improve the patient's functionality, reduce absences from work, and prevent the development of chronic back pain [7,8].

The common treatment for low back pain is to prescribe painkillers, such as acetaminophen or nonsteroidal anti-inflammatory drugs (NSAIDs), while encouraging patients to maintain their daily activities [9,10]. NSAIDs are effective as a short-term treatment for back pain, and are superior to acetaminophen for alleviating pain [11]. Intramuscular application of an NSAID, most commonly diclofenac, is a treatment for acute pain [12]. However, gastrointestinal side effects are common with NSAID use [13]. In recent years, there has been growing concern regarding the safety of cyclooxygenase-2 selective NSAIDs for cardiovascular diseases, especially thrombotic diseases such as acute myocardial infarction, instable angina, stroke, and sudden death [14].

Acupuncture has been widely-used as a method for treating back pain, but there has been controversy about its effects. A systematic review of the literature concluded that acupuncture is effective for pain relief and functional improvement in chronic back pain in the short term, but that, for acute back pain, no evidence of the effectiveness of acupuncture was found [15]. Guidelines for low back pain treatment recommend acupuncture only for chronic back pain [9,10]. There are a variety of ways to administer acupuncture. Motion style acupuncture treatment (MSAT) is different from traditional acupuncture treatments, and is often used in South Korea. Yet, we have not found any clinical trials examining the effectiveness of MSAT. MSAT is similar to traditional acupuncture in that a needle is inserted into an acupuncture point. But, MSAT is novel in that it requires a part of the patient's body to move passively or actively while acupuncture needles are inserted for a certain period of time.

This study is designed to examine the effects of MSAT on acute low back pain with severe disabilities.

## Methods/Design

### Overview

**Figure 1:**
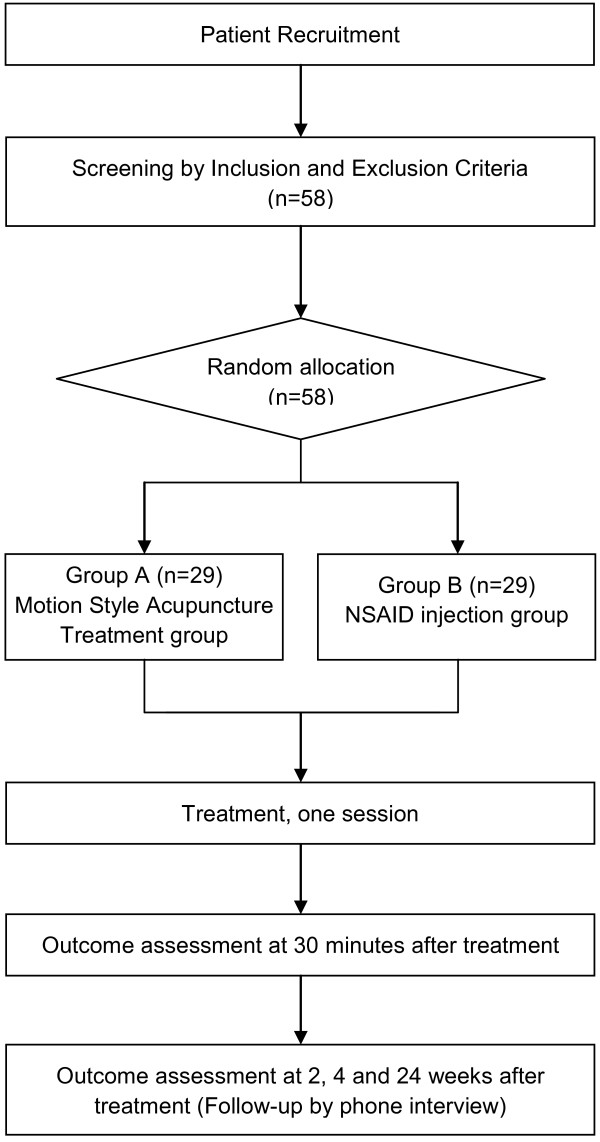
**The Flow of Participants**.

This study is a multicenter, randomized, active-controlled, assessor-blinded trial with two parallel arms. The trial will be conducted in the following two hospitals after obtaining permission from the Institutional Review Boards of the two institutions: Jaseng Hospital of Oriental Medicine in Seoul, Korea and Bucheon Jaseng Hospital of Oriental Medicine in Bucheon, Korea. The volunteers will be randomly divided into two groups (1:1 ratio). The experimental group will receive a single session of MSAT, and the control group will receive a single intramuscular injection of NSAIDs to treat acute low back pain. Thirty minutes after treatment, the subjects will be assessed to determine the outcome of the treatment. Post-treatment follow-up will be performed to measure primary and secondary outcomes at 2, 4, and 24 weeks after treatment (Figure 1).

### Recruitment

We will recruit outpatients who come to Jaseng Hospital of Oriental Medicine in Seoul, Korea and Bucheon Jaseng Hospital of Oriental Medicine in Bucheon, Korea due to acute low back pain. If patients are interested in participating in the study, they will be contacted by a study researcher to determine eligibility in a pre-screening. If the applicant is eligible according to the study criteria, he or she will be examined for eligibility by physicians. And then a study researcher will obtain written informed consent from each eligible participant and administer the baseline questionnaire followed by random allocation of the participant.

### Eligibility

#### Inclusion criteria

• Patients whose low back pain has persisted for less than 4 weeks with or without leg pain.

• Patients with severe disability, defined as an Oswestry Disability Index (ODI) value ≥ 60%.

• Patients who are 20 - 60 years of age.

• Patients who are able to receive a lumbar magnetic resonance imaging (MRI) scan and agree to the procedure.

• Patients who agree to voluntarily participate in this study and sign informed consent.

#### Exclusion criteria

• Patients who have been diagnosed with a serious disease that can cause low back pain (e.g., cancer, vertebral fracture, spinal infection, inflammatory spondylitis, cauda equina compression, or other disqualifying conditions).

• Patients with a chronic disease that could interfere with the effect of the treatment or the interpretation of treatment results (e.g., cardiovascular disease, diabetic neuropathy, fibromyalgia, rheumatoid arthritis, dementia, epilepsy, or other disqualifying conditions).

• Patients with progressive neurological deficit or severe neurological symptoms.

• Patients for whom acupuncture would be inappropriate or unsafe (e.g., hemorrhagic disease, clotting disorders, a history of receiving anticoagulant therapy, severe diabetes with a risk of infection, severe cardiovascular disease, or other disqualifying conditions)

• Patients currently taking corticosteroids, immunosuppressant drugs, psychiatric medicine, or any other medication that may influence the results.

• Patients who had experienced gastrointestinal side effects after taking NSAIDs or are currently being treated for gastrointestinal disease.

• Patients who are currently pregnant or planning to become pregnant.

• Patients who are judged to be inappropriate for the clinical study by the researchers.

### Sample size

The sample size for this clinical study was estimated using the mean difference in numerical rating scale (NRS) for low back pain between the experimental and control groups. We used the NRS as a scale measuring the pain intensity of low back pain. As MSAT is more effective than NSAID injection in previous clinical experience, we set the effect size (Cohen's *d*) > 0.5.

The hypothesis of this study is as follows:

$$H_{0}:\mu_{t}=\mu_{c}\;vs.\;H_{1}:\mu_{t}\neq \mu_{c}$$

*μ_t _*: the mean difference of NRS of the experimental group from baseline

*μ_c _*: the mean difference of NRS of the control group from baseline

Based on previously published results [16], the mean difference of NRS of low back pain in the experimental group from baseline was 3.3, but, as there was no prior existing information on mean difference for the control group, we set the mean difference at 1 based on the experience of a clinical expert. According to these estimations, although the difference between the mean difference of the two groups is 2.3, we conservatively set it as 2. The standard deviation between the two groups was estimated to be 2.5. When a two-tailed test with a test power of 80% and significance level of 5% was applied to the following formula [17], the number of subjects required for each group was 26 people. For a successful study, a total of 58 people, with a 10% dropout rate factored in, is required.

Sample size:

$$n=\frac{2{\left(z_{\alpha ∕2}+z_{1-\beta }\right)}^{2}\sigma^{2}}{{\left(\mu_{t}-\mu_{c}\right)}^{2}}$$

n = the number of subjects required in each group

*μ_t _*- *μ_c _*= 2

*σ *= 2.5

*Z*_*α*/2 _= *Z*_0.05/2 _= 1.96

*Z*_1-*β *_= *Z*_0.8 _= 0.8416

### Randomization

Randomization will be conducted by an expert on statistics who will have no contact with the patients. Random numbers with block randomization will be generated using the SAS version 9.1.3 statistical package (SAS Institute, Cary, NC, USA), and a block size of 6 is used to allocate the two groups (1:1 ratio). The numbers will be kept by a researcher who has no direct contact with the study participants. The randomized numbers will be kept in sealed envelopes, and random allocation will be conducted by opening an envelope as the researcher is informed of a participant's registration at each clinical trial center. In the case of events deemed necessary to code break halfway during trial such as serious adverse events, the researcher will be contacted.

Before the randomization allocation, participants will be informed that they will be assigned to one of the two groups. Random allocation will be performed if a participant is eligible and signs the informed consent form. The subject identification codes will be recorded on case report forms (CRFs) and the randomization table.

### Blinding

This study is designed as a randomized, controlled, assessor-blinded trial. As the experimental group receives acupuncture treatment, namely MSAT, and the control group receives NSAID injection, we are unable to blind both physician and subject to the modality of treatment. Still, assessor-blinding will be achieved by blinding the assessor performing outcome assessment and CRF data entry to the random allocation and treatment of subjects. Statistical analysis will be performed by an independent statistician who is blinded to the identification of each treatment group.

### Treatment protocol

The acupuncture group and the NSAID injection group will each receive a single treatment session.

#### Acupuncture group

The acupuncture treatment will be conducted by oriental medicine doctors who have more than 5 years of clinical experience. These doctors will be required to complete three workshop sessions prior to participating in this study to ensure that the acupuncture is performed as stated in the protocol (see additional file 1 and 2).

First, an assistant stands on each side of the subject with their arms around the patient's shoulders to help the patient stand up. The assistants stand and hold the subject's arms on their shoulders while gently holding one of the subject's hands and holding the subject's waist as if they were lifting. In this position, the practitioner inserts disposable needles to a depth of 10-15 mm at the subject's Pungbu (GV16) and on both sides of Haenggan (LR2) and Gokji (LI11). These acupuncture points were selected according to traditional Chinese medicine theory and previous clinical experiences. When being inserted into GV16 and both sides of LR2, the needles are positioned perpendicular to the body surface. For LI11, the needle is positioned 30 degrees to the body surface. No specific manipulative interventions will be employed in this process. Disposable sterile needles (40 mm × 0.25 mm; Dong-bang Acupuncture, SeongNam, Korea) will be used. Each acupuncture point will be decided on using guidelines based on the World Health Organization Standard Acupuncture Point Locations in the Western Pacific Region [18]. While the needles are still in place, the subject will be asked to walk with the support of the assistants. When the subject's walking improves and the pain is relieved, the practitioner will instruct the assistants to gradually reduce their assistance in three stages and to continue walking with the patient. Once the subject's walking ability improves and the pain is relieved, support is reduced further, and one of the assistants stops supporting the subject. When the subject is able to walk without feeling any severe back pain, the other assistant also stops providing assistance. When the subject is able to walk without a great amount of pain, the treatment is terminated. The procedure takes up to approximately 20 minutes.

If a subject refuses the treatment due to intolerably severe pain during the procedure, the procedure is stopped immediately and the degree of increase in pain and any abnormal reactions are carefully observed and recorded. The total number of subjects who terminate the treatment is recorded.

#### NSAID injection group

Subjects who are assigned to the control group will receive an intramuscular injection of diclofenac sodium (75 mg; KUKJE Pharmaceutical, SeongNam, Korea) in an area of the buttocks where there is no pain. The presence of gastrointestinal side effects or any other side effects will be carefully observed and recorded. In addition, the number of subjects who receive the treatment will be recorded.

### Outcome measurements

When the patients are screened, they will complete questionnaires about their sex, age, height, weight, blood pressure, medical history, and other factors. To determine whether the subjects qualify for the study, their ODI will be calculated through a questionnaire. Their history of back pain, pain intensity, functional status, and other factors will be surveyed. They will also receive a physical examination, X-ray, and lumbar MRI. Evaluations of the patients' back pain will be conducted at baseline and 30 minutes after treatment (Table 1), because the pain relief and improvement in motion due to MSAT appears immediately after the treatment and the maximum plasma concentration for diclofenac is about 10-20 minutes after intramuscular injection. We will also perform additional follow-ups at 2, 4, and 24 weeks and assess the outcome to verify the durability of our treatment. But, we will use telephone interviews to make assessment simpler, and range of motion (ROM) and degree of straight leg raising (SLR) will not be included as outcome measures at post-treatment follow-up for that reason. Researchers who are blinded to the identification of each treatment group and do not participate in the acupuncture treatment will perform the outcome assessment.

**Table 1:** Schedule for data collection and outcome measurement.

Measures	Baseline	30 minutes	2 week	4 week	24 week
Sociodemographic characteristics	●				
History of low back pain	●				
X-ray	●				
Lumbar MRI	●				
NRS for back pain	●	●	●	●	●
NRS for leg pain	●	●	●	●	●
ODI for functional status	●	●	●	●	●
PGIC for global improvement		●			●
ROM of lumbar spine	●	●			
Degrees of SLR	●	●			
Adverse events		●	●	●	●

Abbreviations: MRI, magnetic resonance imaging; NRS, numerical rating scale; ODI, Oswestry disability index; PGIC, patient global impression of change; ROM, range of motion; SLR, straight leg raising

#### Primary outcome measures

Primary outcome refers to the intensity of low back pain, and it will be evaluated using the NRS. Although the NRS is considered a subjective evaluation indicator, it is widely used due to its simplicity. With NRS, a patient chooses one number, ranging from 0 to 10, that best expresses their current level of pain (0 being no pain and 10 being the most excruciating pain the subject has ever experienced) [19,20]. Because the severity of pain can differ at rest and during activity, subjects will be asked this question to reduce errors: "Please indicate the intensity of pain you feel now as you try to move." The NRS for back pain will be obtained at baseline and 30 minutes after treatment and at 2, 4, and 24 weeks after treatment.

#### Secondary outcome measures

The patients' functional status will be evaluated using the ODI questionnaire. It is a 10-item questionnaire developed to evaluate the degrees of disability for lower back pain [21]. Each category is divided into six stages with 0-5 points each. A high number of points indicates severe disability. The accredited Korean version of the ODI questionnaire [22] will be conducted at baseline and 30 minutes and at 2, 4, and 24 weeks after treatment.

To complete a comprehensive evaluation of improvement in back pain and the movement limited by back pain, we will determine the patient global impression of change (PGIC) [19,23]. PGIC is a method for patients to subjectively evaluate their improvement by selecting one of seven stages: 1, very much improved; 2, much improved; 3, minimally improved; 4, no change; 5, minimally worse; 6, much worse; or 7, very much worse. This indicator was originally developed for psychiatry, but it has also been used in other medical areas to assess the improvement of pain. The PGIC will be determined for each patient 30 minutes and 24 weeks after treatment.

Because this study will be conducted with patients who have limited movement due to severe pain, we will check their ROM and degree of SLR to assess the improvement of their movement at baseline and 30 minutes after treatment. The measurement of ROM is reliable (r = 0.94) and valid (r = 0.97) [24], but it is not very responsive (effect size 0.1-0.6) [25]. Also, the measurement of SLR is reliable (intraclass correlation coefficient = 0.95) [26], and the sensitivity is 0.8 (72-97%), the specificity is 0.4(11-66%) [27], but it is not very responsive (effect size = 0.2) [25]. As the responsiveness of ROM and SLR measurement is not high, we decided to use it as a secondary outcome measure instead of a primary outcome measure.

The ROM is checked by measuring the angle between the lumbar spine and a vertical straight line at the patient's full extension and flexion capability. If the measurement is impossible because of pain, the angle is recorded as 0 degrees. To measure degrees of SLR, we will check the leg angle from the patient in a supine position who will then lift each leg up passively with their legs extended. We will measure the angle from the elevated leg to the surface of the floor.

Patients with low back pain may or may not have accompanying leg pain. When leg pain is reported, to assess any improvement in symptoms, the degree of pain is measured for the left and right sides separately by using the NRS. The degree of leg pain might also differ at rest and during activity. Therefore, when the NRS is being evaluated, patients will be asked to answer the question, "Please indicate the intensity of pain you feel now as you are try to move" to minimize errors. The NRS for leg pain will also be checked at baseline and 30 minutes and at 2, 4, and 24 weeks after treatment.

### Statistical analyses

All statistical analyses will be performed in the principle of intention-to-treat analysis using the SAS version 9.1.3 statistical program. For descriptive statistics, normally distributed variables will be expressed as mean and standard deviation. Variables with a skewed distribution or non-parametric variables will be expressed as median and range. For the comparison of the NRS, ODI, ROM and SLR between the two groups, independent t-test will be used, followed by the calculation of effect size (Cohen's *d*) with 95% confidence interval. If there are any differences in age, gender and body mass index between the two groups and the differences are thought to have any influences on the outcome variables, the independent variables will be considered to be covariates and the analysis of covariance (ANCOVA) will be performed. The results will be considered to be statistically significant when P < 0.05.

All adverse events reported during the study will be included in the CRFs; the incidence of adverse events will be calculated. The percentage of subjects with adverse events in each group will be calculated and compared using the Chi-squared test or Fisher's exact test.

### Data handling

Researchers will enter the collected data into the CRFs; unclear or out of range entries and omissions will be recorded on data query forms, which will be returned to the investigational site for resolution. The data from all centers will be pooled and summarized with respect to demographic baseline characteristics, effectiveness, and safety observations.

### Data monitoring

Regular monitoring will be conducted for quality control. Investigators will also convene regularly to discuss practical issues that may be encountered, such as dealing with serious adverse events, revising the protocol, as well as any other important issues raised by the investigators or participants.

### Safety monitoring

The assessment of safety will be based mainly on the frequency of adverse events, which includes all serious adverse events. Information regarding adverse events will be summarized by presenting the number and percentage of participants that experienced adverse events, with the information also categorized according to the body region affected. Any other collected information (e.g., severity or relevance to treatments) will be included in the safety monitoring reports.

### Stopping rules

The trial will stop if the principle investigator believes there is an unacceptable risk of serious adverse events in the groups.

## List of abbreviations used

NSAID: nonsteroidal anti-inflammatory drug; MSAT: motion style acupuncture treatment; ODI: Oswestry Disability Index; MRI: magnetic resonance imaging; NRS: numerical rating scale; CRF: case report form; ROM: range of motion; SLR: straight leg raising; PGIC: patient global impression of change.

## Competing interests

Drs. Joonshik Shin, In-Hyuk Ha, Tae-Gyu Lee, Youngkwon Choi, Byoung-Yoon Park, and Me-riong Kim are employees of Jaseng Hospital of Oriental Medicine during the period of the study. Otherwise, the authors declare that they have no other competing interests.

## Authors' contributions

JSS, IHH, and TGL drafted the protocol and JSS wrote the final manuscript. MSL, YKC, BYP, and MRK contributed to the research design and made critical revisions. JSS is the representative of Jaseng Hospital of Oriental Medicine and participated in the trial design as study director. All of the authors read and approved the final manuscript.

## Pre-publication history

The pre-publication history for this paper can be accessed here:

http://www.biomedcentral.com/1472-6882/11/127/prepub

## Supplementary Material

Additional file 1 **Detailed explanation of method of MSAT**. A text file describing the method, origin, purpose and applications of MSAT. Click here for file

Additional file 2 **Movie file of method of MSAT**. A movie file illustrating the method of MSAT through a demonstration with an actual patient. Click here for file
