# Supplementary material for: Motion style acupuncture treatment (MSAT) for acute low back pain with severe disability: a multicenter, randomized, controlled trial protocol
Source: BMC Complement Altern Med. 2011 Dec 13;11:127. doi: 10.1186/1472-6882-11-127 (PMC3262760; doi:10.1186/1472-6882-11-127)
Supplement: Additional file 1 — Detailed explanation of method of MSAT. A text file describing the method, origin, purpose and applications of MSAT. [file 1472-6882-11-127-S1.DOC]

# Detailed explanation of method of MSAT

## Overview of MSAT

MSAT is a non-traditional acupuncture treatment developed around 1990 by Joon-Shik Shin, a Korean oriental medicine doctor. The technique is highly effective in reducing musculoskeletal pain and increasing limited mobility, and provides almost immediate relief in acute cases with severe pain and restricted motion. MSAT has been used for a wide range of applications, from first-aid treatment for patients suffering from intense pain of acute musculoskeletal origin to various spinal and joint sprains, temporomandibular joint syndrome, frozen shoulder (adhesive capsulitis), gonarthritis, and intervertebral disc herniation.

MSAT is similar to traditional acupuncture in that it places needles in specific meridian acupuncture points and uses manual stimulation of the needles. But, whereas traditional acupuncture encourages the patient to stay still and relax after insertion of the needles, MSAT differs in that it keeps the patient engaged in active or passive action of related body parts for a certain amount of time during acupuncture. The acupuncture points or area of mobilization used in MSAT vary depending on the symptoms or area of pain (i.e., low back pain, neck pain, shoulder pain, knee pain, temporomandibular pain, etc.). The specifics of the type of MSAT used in acute back pain, also known as H-MSAT (Help-Motion Style Acupuncture Treatment) because the patient is “helped” by assistants, are as illustrated below.

## Method of MSAT

The procedure of MSAT as utilized in acute low back pain is as follows. The total time required is approximately 20 minutes.

1. Two assistants stand to each side of the patient and then carefully help the patient to his/her feet and provide support and traction.

2. Acupuncture is applied on GV16, and both sides of LR2 and LI11, consisting of a total of five acupoints in a standing position. The acupuncture needles used are 40 mm*0.25 mm sized disposable needles, which are inserted 10–15mm into the epidermis and underlying muscle.

3. The patient walks slowly, supported by the waist by both assistants, while the needles are still inserted.

4. The patient usually experiences mild to moderate pain and difficulty walking at first, but with time and time-appropriate interventions the initial pain gradually decreases. As the pain alleviates and the patient makes steady progress in his/her difficulty walking, the assistants progressively lessen the level of support and traction in a series of steps as follows until the patient can walk of his/her accord without limitation:

(1) Step 3 (100% support): The assistants support the patient’s weight by drawing themselves as tightly and closely possible to either side of the patient in order to maintain traction at a maximum level.

(2) Step 2 (50% support): The assistant releases some of the patient’s weight and reduce the level of traction to about 50%.

(3) Step 1 (20% support or less): The level of support and traction is brought down to a minimum.

(4) Step 0: The assistant ceases support of the patient who walks shaking his/her arms.

5. When the patient can walk without assistance, he/she is encouraged to walk another 5 minutes after removing the needles in the standing position until the treatment is brought to an end.
